# Efficacy of two commercial synthetic pyrethroids (cypermethrin and deltamethrin) on *Amblyomma variegatum* and *Rhipicephalus microplus* strains of the south-western region of Burkina Faso

**DOI:** 10.1007/s11250-021-02849-2

**Published:** 2021-07-14

**Authors:** Achille S. Ouedraogo, Olivier M. Zannou, Abel S. Biguezoton, Kouassi Yao Patrick, Adrien M. G. Belem, Souaibou Farougou, Marinda Oosthuizen, Claude Saegerman, Laetitia Lempereur

**Affiliations:** 1 Center for Fundamental and Applied Research for Animal and Health (FARAH), Faculty of Veterinary Medicine, ULiège, 4000 Liège, Belgium; 2 Vector-Borne Diseases and Biodiversity Unit (UMaVeB), International Research and Development Centre on Livestock in Sub-Humid Areas (CIRDES), 454 Bobo-Dioulasso 01, Burkina Faso; 3 Research Unit in Epidemiology and Risk Analysis Applied To Veterinary Sciences (UREAR-ULg), Fundamental and Applied Research for Animal and Health (FARAH) , Center, Department of Infectious and Parasitic Diseases, Faculty of Veterinary Medicine, ULiège, 4000 Liège, Belgium; 4 UFR Biosciences, Université Félix Houphouët Boigny, , , BP V34, Abidjan 01, Côte d’Ivoire; 5 Institut du Développement Rural (IDR), Université Nazi BONI, 01 BP 1091 Bobo-Dioulasso 01, Burkina Faso; 6 Communicable Diseases Research Unit, Polytechnic School of Abomey-Calavi, University of Abomey-Calavi, 01 BP 2009 Cotonou, Republic of Benin; 7 Department of Veterinary Tropical Diseases, Faculty Veterinary Science, University of Pretoria, Onderspoort, 0110 South Africa; 8 Laboratory of Parasitology and Parasitic Diseases, Fundamental and Applied Research for Animal and Health (FARAH) Center, Department of Infectious and Parasitic Diseases, Faculty of Veterinary Medicine, ULiège, 4000 Liège, Belgium; 9 Present Address: Federal Public Service Public Health, Food Safety & Environment, President Services, Research Coordination, Place Victor Horta 40, 1060 Brussels, Belgium

**Keywords:** *Amblyomma variegatum*, *Rhipicephalus microplus*, Deltamethrin, Cypermethrin, Burkina Faso

## Abstract

Since 2011, period of the livestock invasion by the cattle tick *Rhipicephalus microplus* in Burkina Faso (BF), tick-control problems were exacerbated. Based on farmer’s reports, most commonly used commercial acaricides were found to be ineffective in Western South part of the country. To investigate the occurrence and extent of such acaricidal ineffectiveness, we performed the standardized larval packet test (LPT) with commercial deltamethrin (vectocid) and cypermethrin (cypertop), on two cattle tick species, the native *Amblyomma variegatum* and the invasive *R. microplus*. The resistance ratios (RR) were computed with susceptible Hounde strain of *Rhipicephalus geigyi* as reference. The *R. microplus* population showed resistance to the two acaricides tested with the highest lethal concentration (LC) values, and different resistance ratios higher than 4 (deltamethrin: RR_50_ = 28.18 and RR_90_ = 32.41; cypermethrin: RR_50_ = 8.79 and RR_90_ = 23.15). In the contrary, *A. variegatum* population was found to be highly susceptible to acaricides tested with low lethal concentrations and resistance ratio values (deltamethrin: RR_50_ = 0.5 and RR_90_ = 0.48; cypermethrin: RR_50_ = 0.68 and RR_90_ = 0.79). These data demonstrate high synthetic pyrethroid resistance in *R. microplus* strain, leading to conclude that the acaricide ineffectiveness in tick populations control remains a concern in BF.

**Supplementary Information:**

The online version contains supplementary material available at 10.1007/s11250-021-02849-2.

## Introduction

About 17 Ixodidae ticks, belonging to the genus *Amblyomma*, *Hyalomma*, and *Rhipicephalus*, have been identified in West Africa (Biguezoton et al. 2016; Diarra et al. 2017; Ouedraogo et al. 2021). These obligate hematophagous arthropods have direct and indirect effects on livestock health and production in most parts of Sub-Saharan Africa (Stachurski 2000). But, by far, the most important indirect impact of ticks is the transmission of hemopathogens, which cause tick-borne diseases (TBD) that can result in high livestock mortalities (De Meneghi et al. 2016). The species *R. microplus* and *A. variegatum* are known to be involved in the main constraints for bovine production system development in the West African context. *Amblyomma variegatum* is an efficient vector of *Ehrlichia ruminantium*, agent of heartwater (cowdriosis). It directly impairs animal growth, tending to heavily reduce milk production (Stachurski 2000; Allsopp 2015). *Rhipicephalus microplus* is the efficient vector of *Babesia bigemina* and *B. bovis*, main agents of bovine babesiosis in tropical region (Adehan et al. 2016; Lempereur et al. 2017). Its introduction into an unaffected area can induce the emergence of its acaricide resistant population (Muhanguzi et al. 2020). For several livestock breeders in West Africa, particularly in BF, tick control is carried out by an increasingly used of acaricidal compounds (Adakal et al. 2013b). The occurrence of the invasive tick species in BF (Adakal et al. 2013a) has increased such tick-control method application, with many cases of misuses reported (Adakal et al. 2013b). As a result, resistance in *R. microplus* population to some commercial acaricidal compounds has been reported (Kande 2014). Since it has been shown (in cattle infestation in BF) that the incidence rate of *R. microplus* significantly increases in the presence of *A. variegatum* and vice versa (Biguezoton et al. 2016), the assessment of the acaricidal resistance of the native species (*A. variegatum*) and invasive species (*R. microplus*) is of paramount importance. For this purpose, the commercial grade synthetic pyrethroids, deltamethrin (vectocid) and cypermethrin (cypertop), widely used in tick control in BF have been tested on larvae of *A. variegatum* and *R. microplus.*

## Materials and methods

### Study area and gorged female collection

**Fig. 1:**
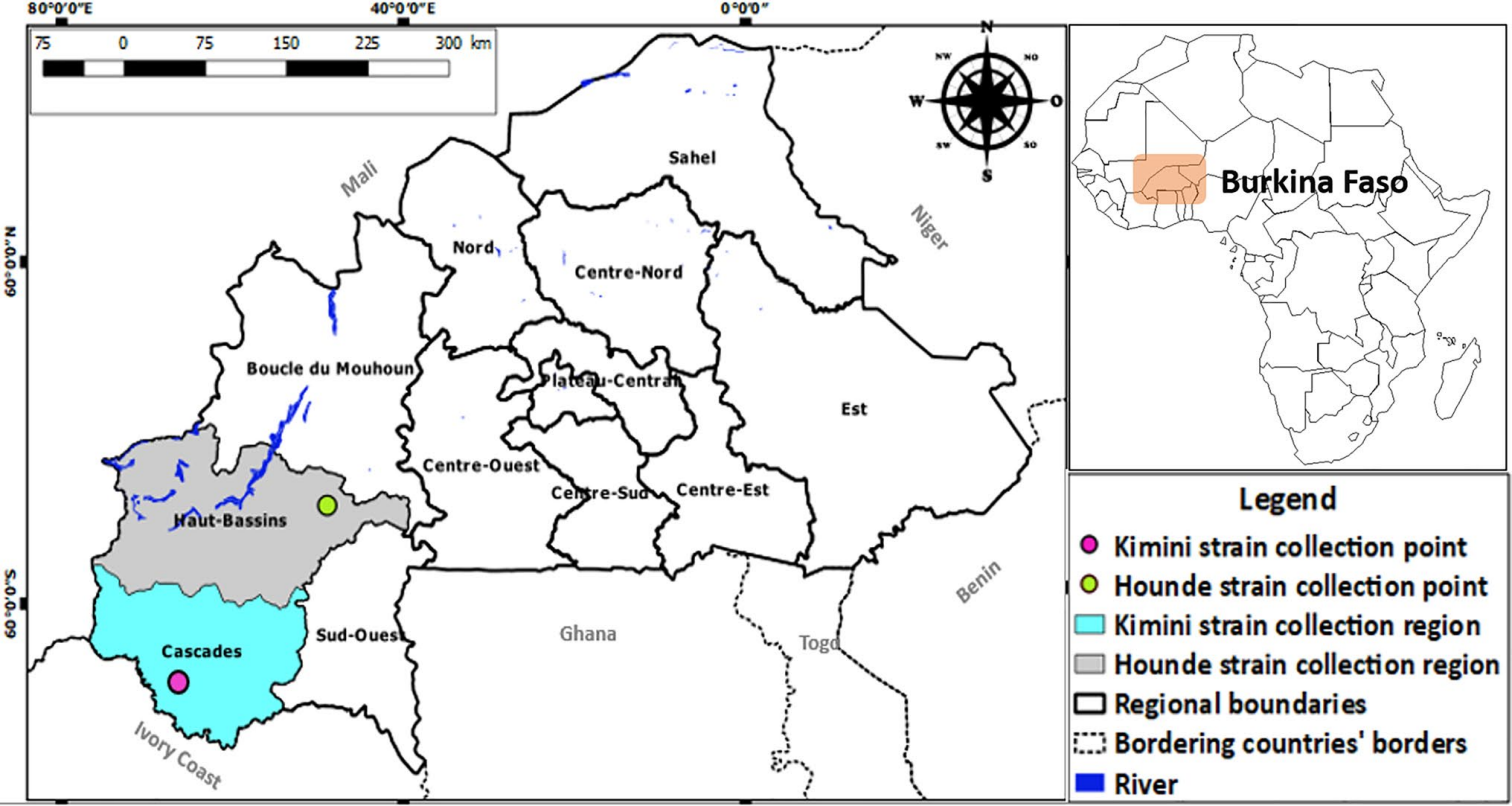
Map showing the geographic location of Burkina Faso, sampling points, and the origin of reference strain

Sampling was carried out during September 2020, in Kimini (N 10.07162; W 4.808), a rural commune located in “Niangoloko,” a department belonging to “Cascades” region, one of the 13 subdivisions of BF territory. This region borders the north of Ivory Coast (Fig. 1). It covers an area of 18,405 km^2^ with about 531,808 habitants active mainly in agriculture and livestock farming. The cattle population was estimated at 654,273 heads in 2013 and represents a source of income for many households (INSD 2018). According to the Adakal et al. (2013b) survey, the most widely acaricide compound used in tick population control in this region is deltamethrin. Farmers involved in this study were requested not to apply any acaricide treatment on cattle at least 2 weeks before the collection date. With the owners’ consent, cattle were kept in lateral decubitus and the whole skin was inspected. All engorged females seen were manually removed, stored in collection jars with lids previously drilled, and closed with compress. Containers were then placed in a plastic bin, with a damp mop on the bottom, in order to allow ticks survival until they reach laboratory at CIRDES (International Research and Development Centre on Livestock in Sub-humid Areas). Once in the lab, ticks were identified, sorted, and about 15–20 live specimens per tick species were placed in an incubator at 27 ± 2 °C with a relative humidity of 85 ± 5% for egg-laying. Eggs obtained were weighed and divided into batches of 0.5 g in different containers. They were then maintained in the same conditions until their hatching.

### Bioassays

The larvae aged between 14 and 21 days were used for the standardized LPT (FAO 2004). The assay was carried out with commercial grade deltamethrin and cypermethrin (respectively Vectocid™ and Cypertop™, LAPROVET, France). For each acaricide, serial dilutions were (Table S1). Different concentrations with a mix ratio of 1 volume olive oil for 2 volumes trichloroethylene (Miller et al. 2002) as solvent were applied. Thereafter, Whatman filter paper was cut into packets of 7.5 × 8.5 cm size and 0.67 ml of each acaricide concentration was applied on each piece of paper filter. The assays were performed in duplicate. For each test, two paper filters were impregnated with solvent (combination of olive oil and trichloroethylene) alone and used as controls. Papers impregnated were placed for 2 h in a fume hood for solvent evaporation. They were then filled with about 100 ticks larvae and incubated at 27 ± 2 °C with 85 ± 5% relative humidity. After 24 h of acaricide exposure, larvae able to move were considered alive, and non-moving one were considered dead. They were counted and mortality rate (death total/total) has been computed for each group/concentration and tick species.

### Reference susceptible strain

The susceptible laboratory strain used as reference was *R. geigyi*. It has been collected at Hounde (N 11.48333; W 3.51667), in the south-western BF in 2005. Engorged females were placed under optimal conditions of temperature (27 ± 2 °C) and relative humidity (85 ± 5%) for egg-laying (Adakal et al. 2013b). This strain is currently maintained in the laboratory of acarology at CIRDES. Over the years, its generations are continuously renewed by artificial infestations on the same cattle breed, Zebu × N’Dama crossbred.

### Data analysis

The package Dose–Response Curves (DRC) was used to perform a non-linear regression analysis of dose-mortality data in R 3.6.3 software. The choice of the model was based on that giving the lowest residual variance through the function mselect (Ritz et al. 2015). The four parameters (b: slope, c: lower value, d: upper value, and e: ED50) were computed with the generalized log-logistic function LL.4 (Ritz et al. 2015). Data were then imported and fitted in GraphPad Prism® 8.0 (GraphPad Software, San Diego, CA, USA) for dose–response curves visualization. The lethal concentrations, LC_50_ and LC_90_, and their 95% confidence intervals (95% CI) were estimated using the effective dose (ED) command. The resistance ratios (ratio between the studied strain ED and that of the reference strain) at 50% (RR_50_) and 90% (RR_90_) of *A. variegatum* and *R. microplus* were computed relatively to the susceptible reference strain *R. geigyi*, with the function ED (Ritz et al. 2015). The resistance status was assessed regarding the reference scale from Jonsson and Hope (2007): a tick population is said to be sensitive to an acaricide when RR < 4; moderately resistant if 4 < RR < 10, and highly resistant when RR > 10.

## Results

### *Amblyomma variegatum* and *R. microplus* resistance status to deltamethrin

**Fig. 2:**
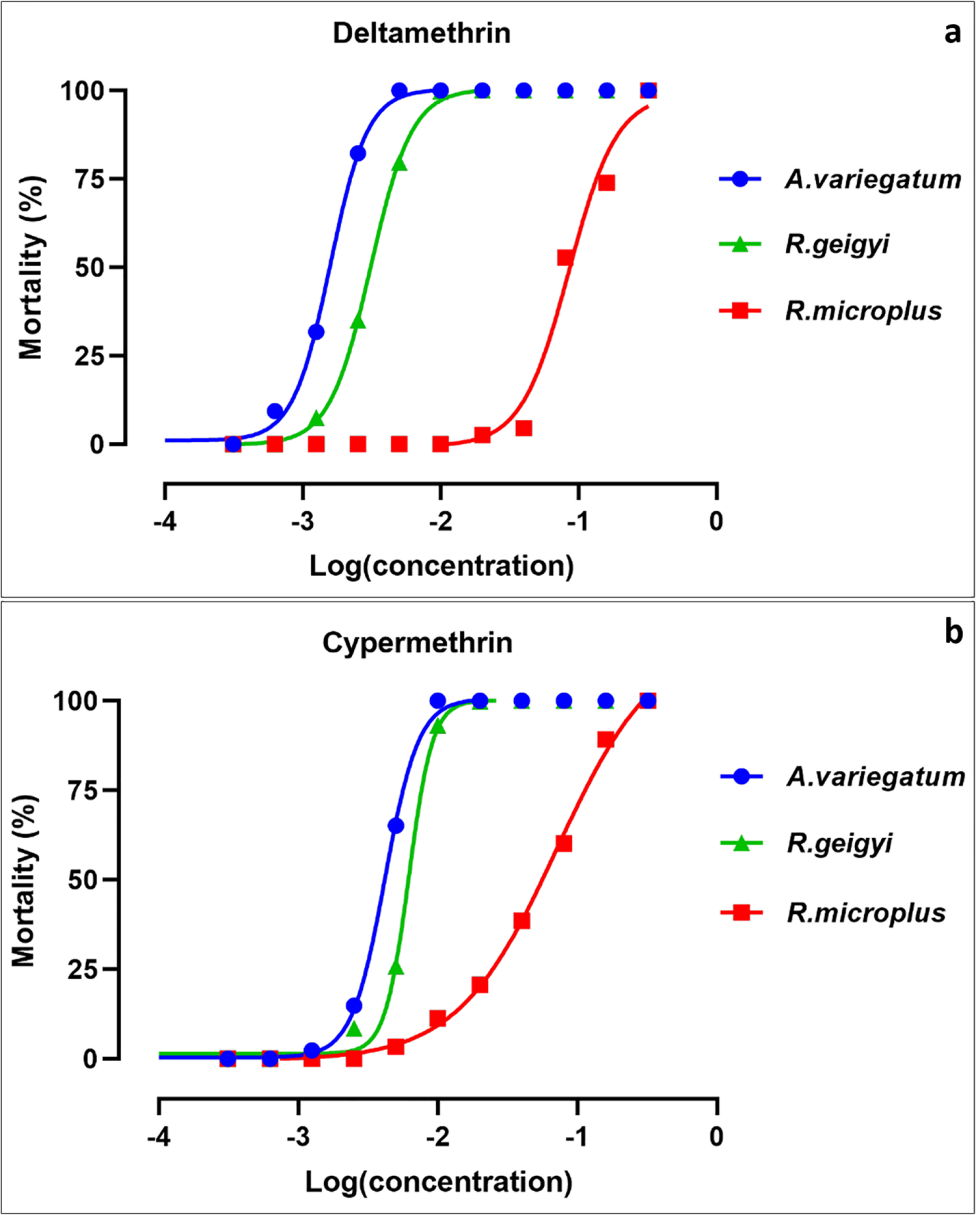
Dose–response curves of sampled *Amblyomma variegatum* and *Rhipicephalus microplus* strains in comparison to the susceptible reference strain *Rhipicephalus geigyi* (Hounde, 2005) when tested with deltamethrin (**a**) and cypermethrin (**b**). Legend: The logarithm of the null (0) concentration (control), considered as 0.0001, (− 5) was excluded from the *X* axis for a best visualization

The strain of *A. variegatum* showed low LC_50_ and LC_90_ values (0.0016 g/l and 0.0031 g/l, respectively) (Table 1). The highest values were found with the *R. microplus* strain (0.0879 g/l and 0.2142 g/l, respectively). The reference strain *R. geigyi* revealed LC_50_ and LC_90_ values lower than the *R. microplus* one (0.0031 g/l and 0.0066 g/l) (Table 1). This is illustrated in Fig. 2a, where *R. geigyi* dose–response curve is located between those of *A. variegatum* (left) and *R. microplus* (right). The curve pattern is also the result of the fairly close slopes (− 2.9, − 3, and − 2.4), reflecting a relatively parallelism. The RR_50_ and RR_90_ values of *A. variegatum* (respectively 0.50 and 0.48) are considerably lower than 4. This leads to conclude to a field strain very susceptible to deltamethrin. In the contrary, *R. microplus* shows RR_50_ and RR_90_ values above 10 (28.18 and 32.41, respectively), indicating a deltamethrin highly resistant strain.

**Table 1:** Lethal concentrations (g/l) of *Amblyomma variegatum* and *Rhipicephalus microplus* and their resistance to deltamethrin and cypermethrin regarding to strain *Rhipicephalus geigyi* as reference strain

Tick species	LC_50_ (95% CI)	LC_90_ (95% CI)	RR_50_ (95% CI)	RR_90_ (95% CI)	Slopes
Deltamethrin					
*R. geigyi*	0.0031 (0.0030–0.00323)	0.0066 (0.0064–0.0068)	–-	–-	− 2.934 ± 0.125
*A. variegatum*	0.0016 (0.0015–0.0016)	0.0031 (0.0001–0.0032)	0.50 (0.48–0.53)	0.48 (0.43–0.53)	− 3.161 ± 0.146
*R. microplus*	0.0879 (0.0776–0.0963)	0.2142 (0.1899–0.2385)	28.18 (24.93–32.41)	32.41 (25.24–45.25)	− 2.437 ± 0.243
Cypermethrin					
*R. geigyi*	0.0062 (0.0059–0.0065)	0.009567 (0.0091–0.010	**–-**	**–-**	− 5.020 ± 0.430
*A. variegatum*	0.0042 (0.0040–0.0044)	0.0076 (0.0073–0.0079)	0.68 (0.64–0.72)	0.79 (0.69–0.90)	− 3.706 ± 0.255
*R. microplus*	0.0547 (0.0484– 0.0610)	0.2258 (0.2012–0.2504)	8.79 (7.83–10.02)	23.15 (18.43–31.10)	− 1.550 ± 0.003

Legend: *R*, *Rhipicephalus*; *A*, *Amblyomma*; *LC*, lethal concentration; *CI*, confidence interval; *RR*, resistance ratio.

### *Amblyomma variegatum* and *R. microplus* resistance status to cypermethrin

Regarding the LC_50_ and LC_90_ values, the same observations were found as in the case of deltamethrin. The values related to the reference strain *R. geigyi* are intermediate to *A. variegatum* (low values, left curve) and *R. microplus* (higher values, right curve) (Table 1, Fig. 2b). However, the dose–response curves did not show any parallelism, as their slopes values are quite dissimilar (− 5; − 3.7; − 1.5) (Fig. 2b). The RR_50_ and RR_90_ values of *A. variegatum* (0.68 and 0.79) were lower than 4 indicating a high susceptibility to cypermethrin. On the opposite, *R. microplus* strain shows RR_50_ value between 4 and 10 (8.79) and a RR_90_ value higher than 10 (23.15), leading to conclude to a cypermethrin moderate to highly resistant strain.

## Discussion

Farmers’ complaints on acaricidal treatment failures, associated with heavy tick infestations in livestock, were the first alert of *R. microplus* tick occurrence in south-western BF (Adakal et al. 2013b). Even if the resistance of *R. microplus* to some acaricidal compounds has been evidenced in BF (Kande 2014), this study represents the first investigation involving the native tick species, *A. variegatum*. As suggested by Adakal et al. (2013b), we assumed that *R. microplus* ticks from Kimini have been introduced in BF from Ivory Coast through transhumance, as hypothesized by local farmers. Thus, the resistance status evidenced here provides an explanation to previous observation on tick-control failures following *R. microplus* introduction in Ivory Coast (Madder et al. 2011). According to FAO (2004), emerging resistance can be suspected when RR_50_ is under 4, while RR_90_ is above, and the field strain slope smaller than the reference strain one. Here, both RR_50_ and RR_90_ values found for *R. microplus* Kimini strain were above 4, both for the commercial grade deltamethrin and cypermethrin. Moreover, the dose–response curve of this strain showed higher slope values than that of the reference strain *R. geigyi*. This leads to conclude to no emerging resistance in *R. microplus* tick population in south-western BF but to an established resistance previously existing in this tick population (Kande 2014). Considering the RR values, *R. microplus* studied strain’s resistance level is in line with that of other strains tested in Brazil with the same compounds by LPT (Mendes et al. 2011; Klafke et al. 2017). Furthermore, some authors (Barré and Uilenberg 2010; Guerrero et al. 2012) suggest that mutations are favored by the rapid generation successions occurring in some parasite species and that could ease the selection of resistant subpopulations in species such as *R. microplus*. Its parasitic phase on the host lasts only 21 days and can have three or four generations per year (Cruz et al. 2020). In contrast, compared to *R. microplus*, the life cycle characteristics of the native tick species *A. variegatum* are different. It is a 3-host tick species, showing a life cycle lasting between about 140 and 270 days (Pegram and Banda 1990; Yonow 1995). The generation change is slow. Therefore, resistant mutations induced by rapid generation successions occurring in the case of *R. microplus* are less unlikely to happen. This could partially explain the high susceptibility of this strain. Moreover, *R. microplus* studied RR values are globally lower than that of some resistant strains reported in Benin (Adehan et al. 2016). This could reflect the various ways and habits in acaricidal compounds use among farmers in the two countries. Indeed, misuses of acaracides, such as the repeated use of the same acaricide, were highlighted in Benin (Achukwi et al. 2001). Furthermore, these results indicate that the aggregation pattern between both tick species on cattle in BF (Biguezoton et al. 2016) did not influence their reaction to the studied acaricides. The native tick species is highly susceptible, while the invasive tick species is resistant to the two acaricides. On contrary to our result, *A. variegatum* was found to be resistant to organophosphates and toxaphene in Ghana, a bordering country of BF (Turkson and Botchey 1999). Even if this finding is not updated, it could suggest a monitoring of tick acaricide resistance, mainly in bordering area in BF, as risks of livestock invasion through transhumance movements remain.

In conclusion, the study provides current resistance status of *A. variegatum* and *R. microplus* regarding commercial grade synthetic pyrethroids in BF. Considering these results, there is a need of a wider investigation on acaricide resistance of the West African *R. microplus* strain and other tick species, for better control strategies of tick infestations. There is also a need to set up a mechanism to monitor the susceptibility status of *A. variegatum* strain to the most commonly used acaricidal compounds, as high selection pressure, through repeated use of the same compounds could lead to acaricidal resistance development.

## Supplementary Information

Below is the link to the electronic supplementary material.

Supplementary file1 (DOCX 14 KB)

## Data Availability

The datasets used during the current study are available from the corresponding author on reasonable request.

## References

[CR1] Achukwi, M.D., Tanya, V.N., Messiné, O., Njongmeta, L.M., 2001. Etude comparative de l’infestation des bovins Namchi (*Bos taurus* ) et Goudali de Ngaoundéré ( *Bos indicus* ) par la tique adulte *Amblyomma variegatum*. Rev. D’élevage Médecine Vét. Pays Trop. 54, 37–41. 10.19182/remvt.9803

[CR2] Adakal, Biguezoton, A., Zoungrana, S., Courtin, F., De Clercq, E.M., Madder, M., 2013a. Alarming spread of the Asian cattle tick *Rhipicephalus microplus* in West Africa—another three countries are affected: Burkina Faso, Mali and Togo. Exp. Appl. Acarol. 61, 383–386. 10.1007/s10493-013-9706-6 10.1007/s10493-013-9706-6 23722233

[CR3] Adakal H, Stachurski F, Chevillon C (2013). Tick control practices in Burkina Faso and acaricide resistance survey in *Rhipicephalus** (**Boophilus**) **geigyi* (Acari: Ixodidae). Exp Appl Acarol.

[CR4] Adehan SB, Abel Biguezoton A, Adakal H, Assogba MN, Zoungrana S, Gbaguidi AM (2016). Acaricide resistance of *Rhipicephalus microplus* ticks in Benin. Afr J Agric Res.

[CR5] Allsopp BA (2015). Heartwater-*Ehrlichia ruminantium* infection. Rev Sci Technol Int Off Epizoot.

[CR6] Barré N, Uilenberg G (2010). Spread of parasites transported with their hosts: case study of two species of cattle tick. Rev Sci Technol.

[CR7] Biguezoton, A., Adehan, S., Adakal, H., Zoungrana, S., Farougou, S., Chevillon, C., 2016. Community structure, seasonal variations and interactions between native and invasive cattle tick species in Benin and Burkina Faso. Parasit. Vectors 9. 10.1186/s13071-016-1305-z 10.1186/s13071-016-1305-z PMC4729031 26819238

[CR8] Cruz BC, de Lima Mendes AF, Maciel WG, dos Santos IB, Gomes LVC, Felippelli G, Teixeira WFP, Ferreira LL, Soares VE, Lopes WDZ, da Costa AJ, de Oliveira GP (2020). Biological parameters for *Rhipicephalus microplus* in the field and laboratory and estimation of its annual number of generations in a tropical region. Parasitol Res.

[CR9] De Meneghi, D., Stachurski, F., Adakal, H., 2016. Experiences in Tick Control by Acaricide in the Traditional Cattle Sector in Zambia and Burkina Faso: Possible Environmental and Public Health Implications. Front. Public Health 4. 10.3389/fpubh.2016.00239 10.3389/fpubh.2016.00239 PMC5101216 27882313

[CR10] Diarra AZ, Almeras L, Laroche M, Berenger J-M, Koné AK, Bocoum Z, Dabo A, Doumbo O, Raoult D, Parola P (2017). Molecular and MALDI-TOF identification of ticks and tick-associated bacteria in Mali. PLoS Negl Trop Dis.

[CR11] FAO., 2004. Resistance management and integrated parasite control in ruminants: guidelines. Module 1. Ticks: Acaricide Resistance: Diagnosis, Management and Prevention. FAO, Rome, Italy pp. 25–77.

[CR12] Guerrero FD, Lovis L, Martins JR (2012). Acaricide resistance mechanisms in *Rhipicephalus** (**Boophilus**) **microplus*. Rev Bras Parasitol Vet.

[CR13] INSD (Institut national de la statistique et de la démographie), 2018. Annuaires statistique de la region des cascades. Septembre 2019

[CR14] Jonsson NN, Hope M (2007). Progress in the epidemiology and diagnosis of amitraz resistance in the cattle tick *Boophilus microplus*. Vet Parasitol.

[CR15] Kande, S., 2014. Évaluation de la résistance des tiques *Rhipicephalus (Boophilus) microplus* (Canestrini, 1888) aux acaricides dans les zones d’introduction en Afrique de l’Ouest (Bénin, Burkina Faso et Côte d’Ivoire). Mémoire de fin de cycle. Univ. Polytechnique Bobo-Dioulasso, 54p

[CR16] Klafke G, Webster A, DallAgnol B, Pradel E, Silva J, de La Canal LH, Becker M, Osório MF, Mansson M, Barreto R, Scheffer R, Souza UA, Corassini VB, dos Santos J, Reck J, Martins JR (2017). Multiple resistance to acaricides in field populations of *Rhipicephalus microplus* from Rio Grande do Sul state, Southern Brazil. Ticks Tick-Borne Dis..

[CR17] Lempereur L, Beck R, Fonseca I, Marques C, Duarte A, Santos M, Zúquete S, Gomes J, Walder G, Domingos A, Antunes S, Baneth G, Silaghi C, Holman P, Zintl A (2017). Guidelines for the Detection of *Babesia* and *Theileria* Parasites. Vector-Borne Zoonotic Dis.

[CR18] Madder M, Thys E, Achi L, Touré A, De Deken R (2011). *Rhipicephalus (Boophilus) microplus*: a most successful invasive tick species in West-Africa. Exp Appl Acarol.

[CR19] Mendes MC, Lima CKP, Nogueira AHC, Yoshihara E, Chiebao DP, Gabriel FHL, Ueno TEH, Namindome A, Klafke GM (2011). Resistance to cypermethrin, deltamethrin and chlorpyriphos in populations of *Rhipicephalus (Boophilus) microplus* (Acari: Ixodidae) from small farms of the State of São Paulo Brazil. Vet Parasitol.

[CR20] Miller RJ, Davey RB, George JE (2002). Modification of the Food and Agriculture Organization Larval Packet Test to Measure Amitraz-Susceptibility Against Ixodidae. J Med Entomol.

[CR21] Muhanguzi D, Byaruhanga J, Amanyire W, Ndekezi C, Ochwo S, Nkamwesiga J, Mwiine FN, Tweyongyere R, Fourie J, Madder M, Schetters T, Horak I, Juleff N, Jongejan F (2020). Invasive cattle ticks in East Africa: morphological and molecular confirmation of the presence of *Rhipicephalus microplus* in south-eastern Uganda. Parasit Vectors.

[CR22] Ouedraogo, A.S., Zannou, O.M., Biguezoton, A.S., Yao, K.P., Belem, A., Farougou, S., Oosthuizen, M., Saegerman, C., Lempereur, L., 2021. Cattle ticks and associated tick-borne pathogens in Burkina Faso and Benin: apparent northern spread of *Rhipicephalus microplus* in Benin and first evidence of *Theileria velifera* and *Theileria annulata*. Ticks Tick-Borne Dis. 101733. 10.1016/j.ttbdis.2021.101733 10.1016/j.ttbdis.2021.101733 33975003

[CR23] Pegram RG, Banda DS (1990). Ecology and phenology of cattle ticks in Zambia: Development and survival of free-living stages. Exp Appl Acarol.

[CR24] Ritz C, Baty F, Streibig JC, Gerhard D (2015). Dose-Response Analysis Using r. PLOS ONE.

[CR25] Stachurski F (2000). Invasion of West African cattle by the tick *Amblyomma variegatum*. Med Vet Entomol.

[CR26] Turkson PK, Botchey M (1999). Acaricide resistance in the cattle tick, *Amblyomma variegatum*, in the coastal savanna zone of Ghana. Ghana J Agric Sci.

[CR27] Yonow T (1995). The life-cycle of *Amblyomma variegatum* (Acari: Ixodidae): a literature synthesis with a view to modelling. Int J Parasitol.

