# Supplementary material for: Efficacy of two commercial synthetic pyrethroids (cypermethrin and deltamethrin) on Amblyomma variegatum and Rhipicephalus microplus strains of the south-western region of Burkina Faso
Source: Trop Anim Health Prod. 2021 Jul 14;53(3):402. doi: 10.1007/s11250-021-02849-2 (PMC8277628; doi:10.1007/s11250-021-02849-2)
Supplement: Supplementary file 1 — Supplementary file1 (DOCX 14 KB) [file 11250_2021_2849_MOESM1_ESM.docx]

**Table_S1 :** **Summary of synthetic pyrethroids tested and dilution series applied**

| Tick species | Acaricides tested (95 % purity) | Strains (GPS coordonates) | Dilution series (g/l) |
| --- | --- | --- | --- |
| *R. geigyi* | Deltamethrin | Hounde (N 11.48333 ; W 3.51667) | 0-0.00125-0.0025-0.005-0.01-0.02-0.04-0.08-0.16-0.32 |
|  | Cypermethrin |  |  |
| *A.variegatum* | Deltamethrin | Kimini (N 10.07162; W 4.808) | 0-0.0003125-0.000625-0.00125-0.0025-0.005-0.01-0.02-0.04-0.08-0.16-0.32 |
|  | Cypermethrin |  |  |
| *R. microplus* | Deltamethrin | Kimini (N 10.07162; W 4.808) |  |
|  | Cypermethrin |  |  |

Legend: R: *Rhipicephalus*, A: *Amblyomma*, BF: Burkina Faso.
